# Reliability of a Newly-Developed Immunochromatography Diagnostic Kit for Pandemic Influenza A/H1N1pdm Virus: Implications for Drug Administration

**DOI:** 10.1371/journal.pone.0050670

**Published:** 2012-11-30

**Authors:** Tadahiro Sasaki, Ritsuko Kubota-Koketsu, Michihiro Takei, Tatsuo Hagihara, Shinichi Iwamoto, Takuya Murao, Kazuo Sawami, Daizou Fukae, Masahiro Nakamura, Eiichi Nagata, Akira Kawakami, Yuko Mitsubayashi, Masafumi Ohno, Yasuo Uehara, Takashi Fukukawa, Yuta Kanai, Mieko Kosaka, Kazuyoshi Ikuta

**Affiliations:** 1 Department of Virology, Research Institute for Microbial Diseases, Osaka University, Suita, Osaka, Japan; 2 Osaka Higashinari-ku Medical Association, Higashinari-ku, Osaka, Japan; 3 Alfresa Pharma Corporation, Ibaraki, Osaka, Japan; Fudan University, China

## Abstract

**Background:**

For the diagnosis of seasonal influenza, clinicians rely on point-of-care testing (POCT) using commercially available kits developed against seasonal influenza viruses. However, POCT has not yet been established for the diagnosis of pandemic influenza A virus (H1N1pdm) infection due to the low sensitivity of the existing kits for H1N1pdm.

**Methodology/Principal Findings:**

An immunochromatography (IC) test kit was developed based on a monoclonal antibody against H1N1pdm, which does not cross-react with seasonal influenza A or B viruses. The efficacy of this kit (PDM-IC kit) for the diagnosis of H1N1pdm infection was compared with that of an existing kit for the detection of seasonal influenza viruses (SEA-IC kit). Nasal swabs (n = 542) were obtained from patients with flu-like syndrome at 13 clinics in Osaka, Japan during the winter of 2010/2011. Among the 542 samples, randomly selected 332 were further evaluated for viral presence by reverse transcriptase polymerase chain reaction (RT-PCR). The PDM-IC kit versus the SEA-IC kit showed higher sensitivity to and specificity for H1N1pdm, despite several inconsistencies between the two kits or between the kits and RT-PCR. Consequently, greater numbers of false-negative and false-positive cases were documented when the SEA-IC kit was employed. Significant correlation coefficients for sensitivity, specificity, and negative prediction values between the two kits were observed at individual clinics, indicating that the results could be affected by clinic-related techniques for sampling and kit handling. Importantly, many patients (especially influenza-negative cases) were prescribed anti-influenza drugs that were incongruous with their condition, largely due to physician preference for patient responses to questionnaires and patient symptomology, as opposed to actual viral presence.

**Conclusions/Significance:**

Concomitant use of SEA-IC and PDM-IC kits increased the likelihood of correct influenza diagnosis. Increasing the credibility of POCT is anticipated to decrease the inappropriate dispensing of anti-influenza drugs, thereby minimizing the emergence of drug-resistant H1N1pdm strains.

## Introduction

Swine-origin pandemic influenza A virus (subtype H1N1pdm) emerged in April, 2009 and rapidly spread across the globe, becoming one of the most common human influenza A viruses in the world [1]. Seasonal influenza virus A subtype H1N1, by contrast, had all but disappeared from most countries by the 2009/2010 winter influenza season. However, H1N1pdm was replaced by a mixed population of H1N1pdm and seasonal influenza A subtype H3N2 during the 2010/2011 winter influenza season [2].

The H1N1pdm virus contains a triple-reassortant genome that includes a combination of avian, human, and swine influenza virus gene segments. The H1N1pdm genome encodes polymerase basic protein 2 (PB2) and polymerase subunit A (PA), both derived from the North American avian lineage; polymerase subunit B1 (PB1), derived from human seasonal influenza A H3N2; neuraminidase protein (NA) and matrix (M) proteins, derived from the Eurasian swine lineage; and hemagglutinin (HA), nucleoprotein (NP), and nonstructural (NS) proteins, derived from the North American classical swine lineage [3].

Based on epidemiological data from Mexico, where the estimated case fatality ratio in 2009 was 1.2% overall and 5.5% among individuals over 60 years of age [10], [11], [12], as well as on animal studies of influenza virus infection [13], the pathogenicity of H1N1pdm was initially thought to be relatively high. Subsequent estimates of case fatality ratios were, however, significantly lower than the initial estimates [14], [15]. In particular, the case fatality ratio in Japan was only 0.1% [16], [17]. It is likely that one of the reasons for the low case fatality ratio is an established system in Japan for the rapid diagnosis of influenza virus and the subsequent administration of anti-influenza drugs.

Several groups are at elevated risk for H1N1pdm infection, including pregnant women, individuals with diabetes, and the obese, elderly, and very young [4]. However, rapid diagnosis of the infection followed by administration of appropriately prescribed anti-viral drugs considerably attenuates disease severity and duration, even in patients belonging to these high-risk groups. The majority of H1N1pdm strains are susceptible to oseltamivir (Tamiflu), although H1N1pdm oseltamivir-resistant strains are on the rise and account for 0.5–1.0% of all cases in most countries [5]–[8]. On the other hand, the H1N1pdm virus carries an S31N mutation in the M2 gene and is therefore resistant to treatment with adamantanes [9].

Rapid immunologic diagnosis of H1N1pdm was attempted soon after its emergence in 2009 by using immunochromatography (IC) test kits previously developed for the detection of seasonal influenza viruses (SEA-IC kits). Nonetheless, these SEA-IC kits could not reliably differentiate H1N1pdm from seasonal influenza A viruses H1N1 and H3N2. Indeed, subsequent analysis via reverse transcriptase polymerase chain reaction (RT-PCR) indicated that existing SEA-IC test kits showed significantly low sensitivity for H1N1pdm [18], [19], [20], [21], [22], [23], [24], [25], [26]. Consequently, we developed murine monoclonal antibodies that were specific for H1N1pdm, which exhibited no cross-reactivity with seasonal influenza A (H1N1 or H3N2) or seasonal influenza B viruses. These monoclonal antibodies were then employed to develop a new IC test kit for the high-sensitivity detection of H1N1pdm [27].

Our previous research with clinical samples confirmed the efficacy of the newly-developed H1N1pdm IC test kit (PDM-IC kit) [27]. This work included H1N1pdm-confirmed clinical samples that were obtained during the 2009/2010 winter influenza season from patients at a single clinic in Osaka, Japan. Clinical samples containing other influenza subtypes obtained before 2009 were used as controls to evaluate the specificity of the PDM-IC test kit.

In the current study, the PDM-IC test kit was evaluated for the rapid diagnosis of H1N1pdm infection using clinical samples obtained during the 2010/2011 winter influenza season in Osaka, Japan. A total of 542 clinical samples collected from patients at 13 clinics were involved in the evaluation. The PDM-IC kit was assessed relative to a previously-developed SEA-IC kit for its accuracy and limitations of sensitivity, specificity, positive predictive values (PPVs), and negative predictive values (NPVs). Subsets of the samples were also subjected to RT-PCR and virus isolation (VI) analyses. The data were then compared with clinical data regarding symptoms and drug prescriptions so as to determine the general applicability and reliability of rapid diagnosis kits for point-of-care testing (POCT) at clinics.

## Materials and Methods

### IC Rapid Test Kits

The Prime Check Flu (H1N1) 2009 (Alfresa Pharma Corporation, Osaka, Japan) for the rapid detection of the NP protein derived from H1N1pdm [27] was employed in this study and was referred to as the “PDM-IC kit”. An IC kit currently in use for the rapid detection of NP proteins derived from seasonal influenza A and B viruses (Check Flu A+B kit; Alfresa Pharma Corporation, Osaka, Japan) was employed as the control. This kit was referred to as the “SEA-IC kit”. Importantly, the same solution for the suspension of swab samples could be used for tests performed with both kits.

### Sample Collection

Samples of nasal or nasopharyngeal fluid were collected from patients with influenza-like symptoms (n = 542) at 13 clinics (termed “A” through “M” clinics) under the guidance of the Higashinari-ku Medical Association in Osaka, Japan during the winter of 2010–2011 (November, 2010 through April, 2011). Two swabs were taken from each patient; one sample was tested using the PDM-IC and SEA-IC kits, and the other was tested using RT-PCR and/or VI.

### Viruses and Cells

The following virus strains were used: A/Suita/01/09 (H1N1pdm virus); A/New Caledonia/20/99 (seasonal influenza A virus H1N1); A/Wyoming/2/03 (seasonal influenza A virus H3N2); and B/Malaysia/2506/04 (seasonal influenza B virus). Madin-Darby canine kidney (MDCK) cells were used for propagation of the viruses. The MDCK cells were maintained in Minimum Essential Medium (MEM) supplemented with 10% fetal bovine serum (FBS) in a humidified incubator (5% CO_2_/95% air) at 37°C.

### RT-PCR Analysis

RT-PCR-based analysis for the highly sensitive detection of viral RNA and genotyping of influenza viruses in the clinical specimens was conducted as described previously [27]. For RT-PCR analysis, 332 samples were randomly selected from the initial 542 swab samples. Briefly, viral RNA was extracted from the clinical samples using a QIAamp® Viral RNA Mini kit (QIAGEN, Tokyo, Japan). RT-PCR was then performed with a QIAGEN OneStep RT-PCR kit for H1N1pdm and a SuperScriptTM III One-Step RT-PCR System with Platinum® Taq High Fidelity (Invitrogen, Carlsbad, CA) for the seasonal influenza viruses. The RT-PCR conditions were as follows: 50°C for 30 min and 94°C for 3 min, followed by 40 cycles at 94°C for 30 sec, 54°C for 30 sec, 72°C for 30 sec, and a final extension at 72°C for 7 min.

### VI Analysis

For VI analysis, 263 samples were further selected from the 332 swab samples that were subjected to RT-PCR. The 263 samples included all inconsistent cases (n = 79) between the two kits or between the kits and RT-PCR. The additional 184/263 samples were randomly selected from the original 332 RT-PCR samples. To isolate the influenza viruses from the clinical samples, MDCK cells were inoculated with clinical specimens and incubated at 37°C in Dulbecco’s Modified Eagle Medium (DMEM) containing Nutrient Mixture F-12 (DMEM/F12, Invitrogen) and 1% v/v Antibiotic-Antimycotic liquid (Invitrogen), 0.4% w/v bovine serum albumin, and 2 µg/mL Trypsin Acetylated from Bovine Pancreas, Type V-S (Sigma-Aldrich, St. Louis, MO). Cell were incubated for 3 weeks, or until cytopathic effects were observed.

### Virus Identification by Peroxidase-anti-peroxidase (PAP) Staining

Isolated viruses were identified by PAP staining using two influenza virus A subtype-specific murine monoclonal antibodies, C179 (H1-specific) and F49 (H3-specific), and the influenza virus B HA-specific murine monoclonal antibody, 7B11 (unpublished, Yoshinobu Okuno, Osaka Prefectural Institute of Public Health, Osaka, Japan), as described previously [27]. Briefly, MDCK cells in MEM were inoculated with the isolated viruses and incubated at 37°C for 16 h. After fixation with absolute ethanol, the cells were incubated with the monoclonal antibodies described above followed by rabbit anti-mouse immunoglobulin (1∶1,000; Organon Teknika, Malvern, PA). The cells were treated successively with goat anti-rabbit immunoglobulin G antibody (1∶500; Organon Teknika) and PAP (rabbit anti-peroxidase) complex (1∶5,000; Organon Teknika). Finally, a peroxidase reaction was conducted by incubating the cells with 3,3′-diaminobenzidine tetrahydrochloride substrate for 5 min at room temperature. The stained cells were observed under an inverted optical microscope.

### Statistical Analysis

This study used the results of RT-PCR as the “gold standard”. Based on RT-PCR, the results of the test kits were divided into four groups, i.e., “true-positive (TP)”, “true-negative (TN)”, “false-positive (FP)”, and “false-negative (FN)” cases. Results of the SEA-IC kit were divided into TP, TN, FP, and FN cases, as follows: TP (“A+B−” with seasonal H1N1, H1N1pdm, or H3N2 by RT-PCR; or “A−B+” with influenza B virus by RT-PCR); TN (“A−B−” with “−” by RT-PCR); FP (“A+B−” with “−” by RT-PCR); and FN (“A−B−” with seasonal H1N1, H1N1pdm, H3N2, or influenza B virus by RT-PCR). Results of the PDM-IC kit were divided into TP, TN, FP, and FN cases, as follow: TP (“pdm+” with H1N1pdm by RT-PCR); TN (“pdm− “ with “−”, seasonal H1N1, H3N2, or influenza B virus by RT-PCR); FP (“pdm+” with “−”, seasonal H1N1, H3N2, or influenza B virus by RT-PCR); and FN (“pdm−” with H1N1pdm by RT-PCR). Statistical analysis was performed for sensitivity values, specificity values, positive predictive values (PPV), and negative predictive values (NPV) which were calculated according to the following formulas: sensitivity = TP/(TP+FN); specificity = TN/(FP+TN); PPV = TP/(TP+FP); and NPV = TN/(FN+TN). All results were assessed by correlation analysis, Student’s t-test, or the chi-square test, performed using SPSS version 18 (SPSS, Chicago, IL). The level of statistical significance was set at P<0.05.

### Ethics

The research protocol for the collection of human samples was approved by the Ethics Committee of the Research Institute for Microbial Diseases, Osaka University, Japan. Informed consent was obtained from all patients in writing before enrollment in the study.

## Results

### Patient Background

Swab samples (n = 542 duplicate samples) were simultaneously collected from 542 patients with influenza-like symptoms presenting at 13 clinics (clinics “A” through “M”) within Higashinari-ku, Osaka, Japan from November, 2010 through April, 2011 (Table 1). The study included 271 males and 266 females (gender unknown in five cases), with a mean age of 30.5 years (range, 0–88 years; age unknown in 14 cases) and an average of 1.2±1.2 days between the onset of clinical signs and sampling (onset time unknown in 18 cases) (Table 1).

### Reliability of Data Yielded by Rapid Test Kits, RT-PCR, and VI Studies

Of the two swab samples collected from each patient, one sample was immediately tested using the PDM-IC kit and the SEA-IC kit, and the other was kept at −80°C and subsequently used for RT-PCR and VI analyses. The PDM-IC and SEA-IC kits were designed for the rapid detection of NP proteins derived from the H1N1pdm virus and the seasonal influenza A and B viruses, respectively.

**Figure 1:**
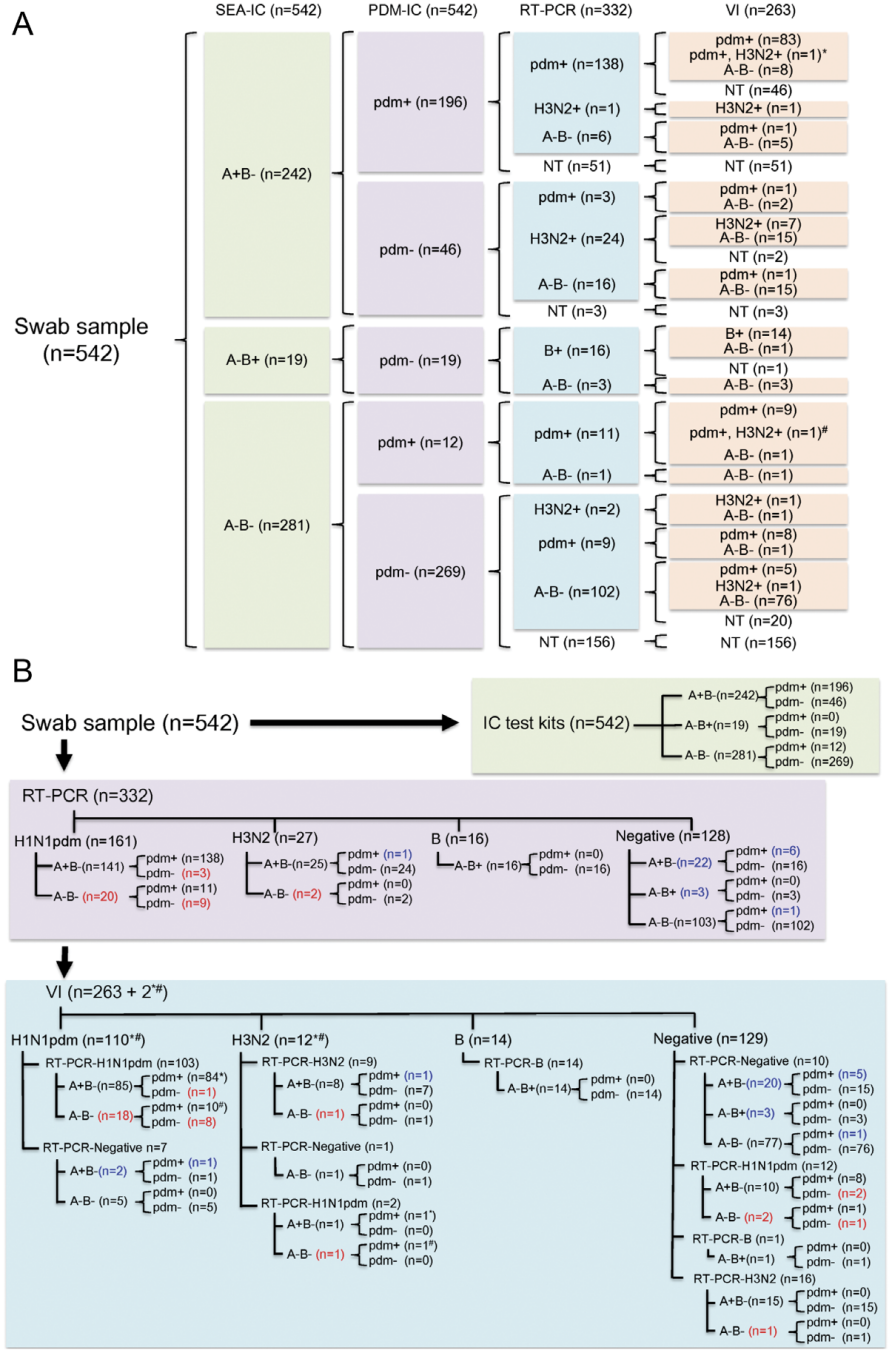
Summarized results of 524 swab samples analyzed by POCT with two IC test kits (SEA-IC and PDM-IC) kits, RT-PCR, and VI. A, a total of 542 swab samples were subjected to POCT at 13 clinics. Subsequently, 332 of the 542 samples were subjected to RT-PCR, and 263 of the 332 samples analyzed by RT-PCR were subjected to VI. The SEA-IC results for influenza A virus (“A+B−”) or influenza B virus (“A−B+”), as well as the PDM-IC results for pandemic influenza A virus H1N1pdm(“pdm+” and “pdm−”), are shown. Both H1N1pdm and H3N2 were isolated from two samples, as shown by the (*) and (#). B, The results of (A) are summarized for the individual diagnostics procedures (IC test kits, 542 samples; RT-PCR, 332 samples; and VI, 263 samples). The samples that yielded false-positive and false-negative results following analysis with the SEA-IC and PDM-IC kits (based on the RT-PCR results as the gold standard) are shown in blue and red, respectively.

The results from the SEA-IC kit were as follows: 44.7% (242/542) of the cases tested positive for influenza A virus; 3.5% (19/542) of the cases tested positive for influenza B virus; and the remaining 51.8% (281/542) of the cases tested negative for either influenza A or B virus (Figure 1A). The PDM-IC kit identified 38.4% (208/542) of the cases as positive for the H1N1pdm virus (Figure 1A). To evaluate the sensitivity and specificity of the two IC test kits for H1N1pdm, 332 randomly selected swab samples out of the 542 original swab samples were analyzed by RT-PCR as a “gold standard”. The results for the influenza virus-positive cases are summarized in Figure 1B according to the data obtained from each type of IC kit (PDM-IC versus SEA-IC), and RT-PCR.

**Table 1:** Background information for patients with influenza-like symptoms from whom clinical specimens were collected.

Clinic	Number of samples	Gender			Age		Specimen collection	
		Male	Female	Unknown^a^	Mean years (Range)	Unknown^a^	Days after onset	Unknown^a^
A	40	18	21	1	26.4 (1–69)	1	0.8±0.6	1
B	10	6	4	0	28.3 (10–51)	0	1.2±0.4	1
C	79	39	40	0	29.7 (9–76)	0	1.0±0.7	0
D	70	28	40	2	24.5 (2–62)	2	1.1±0.9	3
E	17	6	11	0	30.8 (4–88)	2	1.9±2.8	0
F	39	16	23	0	27.4 (3–63)	1	1.1±0.7	1
G	30	18	12	0	31.8 (2–73)	0	1.5±0.7	0
H	48	24	23	1	25.5 (0–86)	0	0.8±0.7	1
I	38	21	16	1	34.1 (12–62)	0	1.3±0.9	2
J	39	20	19	0	33.6 (9–60)	7	1.0±0.6	6
K	22	11	11	0	31.3 (20–45)	0	1.7±0.6	0
L	48	30	18	0	40.7 (21–78)	1	1.7±1.8	0
M	62	34	28	0	34.7 (2–87)	0	1.5±1.5	3
Total	542	271	266	5	30.5 (0–88)	14	1.2±1.2	18

a Information could not be obtained from the answers to the questionnaires.

The results of the RT-PCR analysis were as follows: 48.5% (161/332) of the cases tested positive for H1N1pdm; 8.1% (27/332) of the cases tested positive for seasonal influenza A virus H3N2; 4.8% (16/332) of the cases tested positive for influenza B virus; and 38.6% (128/332) of the cases tested negative for any type of influenza virus (Figure 1). No seasonal H1N1-positive cases were detected.

**Figure 2:**
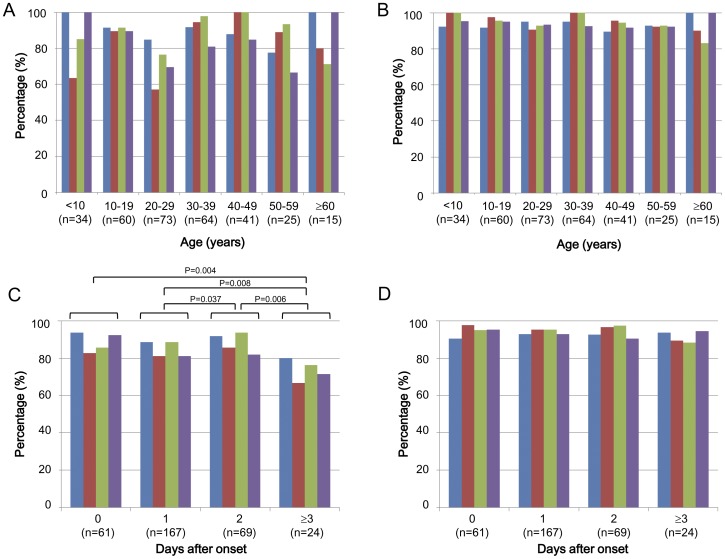
Effect of patient age and duration from disease onset to collection of samples on rapid test kit results. The SEA-IC test kit (A, C) and the PDM-IC test kit (B, D) were independently evaluated for sensitivity (blue), specificity (red), PPV (green), and NPV (purple) according to patient age (from <10 to ≥60 years old) (A, B) and duration from disease onset to sampling (1, 2, and ≥3 days) (C, D). P values in (C) indicate significant differences between groups. The 332 samples with information regarding patient age were used for (A) and (B), and the 321 samples with information regarding the number of days after disease onset were used for (C) and (D).

The SEA-IC kit showed 89.2% sensitivity, 80.5% specificity, 87.9% PPV, and 82.4% NPV based on the data for influenza virus-positive cases obtained by RT-PCR (332 cases) (Table 2). By contrast, the PDM-IC kit showed 92.5% sensitivity, 95.3% specificity, 94.9% PPV, and 93.1% NPV among 332 cases (Table 2). Interestingly, the SEA-IC kit and PDM-IC kit results from individual clinics showed significant correlations in terms of sensitivity, specificity, and NPV (correlation coefficients 0.706, 0.657, and 0.784; P values 0.007, 0.020, and 0.003, respectively). These results suggest that the rapid IC kit scores could be affected by clinic-related techniques for swab sampling and kit handling. The SEA-IC kit results showed a tendency toward variations in sensitivity, specificity, PPV, and NPV between different age groups (<10, 10–19, 20–29, 30–39, 40–49, 50–59, and ≥60 years) (Figure 2A), while the PDM-IC kit results showed higher overall scores and no clear variations between age groups (Figure 2B). Thus, the PDM-IC kit tended to yield stable results regardless of patient age.

**Table 2:** Evaluation of rapid test kits for POCT at each clinic.

Clinic	Sensitivity		Specificity		PPV^a^		NPV^b^	
	SEA-IC	PDM-IC	SEA-IC	PDM-IC	SEA-IC	PDM-IC	SEA-IC	PDM-IC
A (n = 15)	90.9	83.3	100	100	100	100	80.0	90.0
B (n = 10)	50.0	60.0	100	100	100	100	57.1	71.4
C (n = 56)	100	100	89.3	94.7	90.3	90.0	100	100
D (n = 60)	82.2	90.5	100	94.4	100	97.4	65.2	81.0
E (n = 11)	100	75.0	80.0	100	85.7	100	100	87.5
F (n = 16)	100	100	100	85.7	100	90.0	100	100
G (n = 21)	88.2	93.3	25.0	66.7	83.3	87.5	33.3	80.0
H (n = 28)	90.9	87.5	76.5	100	71.4	100	92.9	95.2
I (n = 16)	100	100	NA^c)^	100	75.0	100	NA	100
J (n = 15)	75.0	60.0	85.7	100	85.7	100	75.0	83.3
K (n = 16)	100	100	66.7	100	92.9	100	100	100
L (n = 32)	95.5	100	60.0	92.3	84.0	95.0	85.7	100
M (n = 36)	71.4	91.7	81.8	100	71.4	100	81.8	96.0
Total (n = 332)	89.2	92.5	80.5	95.3	87.9	94.9	82.4	93.1
Correlationcoefficient	0.706		0.657		0.180		0.784	
P value	0.007		0.020		0.555		0.003	

a Positive predictive value.

b Negative predictive value.

c Not applicable.

Further analysis regarding sampling time after the onset of clinical signs was next performed. The accuracy of the results obtained using the SEA-IC kit significantly decreased for samples collected on the third or more day after onset (4.2±2.2 days) compared with those collected on the previous days (Figure 2C). This was in contrast to the results obtained using the PDM-IC kit, which showed similar scores for all of the samples collected during the study period, even at the third or more days after the onset of clinical symptoms (Figure 2D). Consequently, although the exact reason for the variable results among the individual age groups (Figure 2A) is not clear, it seems to be due, at least in part, to the duration from disease onset to POCT. In this regard, the SEA-IC kit compared with the PDM-IC kit afforded more false-positive cases (as assessed by specificity and PPV) and false-negative cases (as assessed by sensitivity and NPV) among the RT-PCR-positive samples obtained from patients with a longer duration from disease onset to POCT. For example, an increased number of false-positive cases was observed for patients aged 20–29 years and ≥60 years with a duration from disease onset to POCT of 2.0±1.65 and 2.50±0.71 days, respectively. Similarly, an increased number of false-negative cases was observed for patients aged 20–29 years and 40–49 years with a duration from disease onset to POCT of 1.71±0.76 and 1.67±1.15 days, respectively (Table S1).

As summarized in Figure 1, the two IC test kits afforded inconsistent results in several cases (2.2%, 12/542), the two IC test kits afforded inconsistent results: 11 samples tested negative for influenza viruses by SEA-IC, but tested positive for H1N1pdm by PDM-IC and RT-PCR; and one sample tested negative for influenza viruses by SEA-IC and RT-PCR, but tested positive for H1N1pdm by PDM-IC. Furthermore, several inconsistent cases among the 332 samples subjected to RT-PCR were also identified between the SEA-IC kit and RT-PCR (n = 47: 22 false-negative cases consisting of 20 “A−B−”/“H1N1” cases by SEA-IC/RT-PCR and two “A−B−”/“H3N2” by SEA-IC/RT-PCR; and 25 false-positive cases consisting of 22 “A+B−”/“−”by SEA-IC/RT-PCR and three “A−B+”/“−” by SEA-IC/RT-PCR); as well as between the PDM-IC kit and RT-PCR (n = 20: 12 false-negative cases consisting of “pdm−”/“H1N1pdm” by PDM-IC/RT-PCR; and eight false-positive cases consisting of one “pdm+”/“H3N2” by PDM-IC/RT-PCR and seven “pdm+”/“−” by PDM-IC/RT-PCR).

Further, the 263 samples, including the above-described samples showing inconsistent results between two IC test kits (n = 12), between the SEA-IC kit and RT-PCR (n = 47), and between the PDM-IC kit and RT-PCR (n = 20), were analyzed by VI. As summarized in Figure 1, the results of the VI analysis indicated that 41.1% (108/263) of the cases were positive for H1N1pdm; 3.8% (10/263) of the cases were positive for H3N2; 0.8% (2/263) of the cases were positive for both H1N1pdm and H3N2; and 5.3% (14/263) of the cases were positive for influenza B virus. The remaining 49.0% (129/263) of the cases tested negative for influenza virus infection. Thus, there were several cases that tested positive by VI, but negative by RT-PCR (n = 8; seven for H1N1pdm and one for H3N2), and vice versa (n = 29; 12 for H1N1pdm, 16 for H3N2, and one for influenza B virus).

### Relationship between Clinical Symptoms and IC Test Kit Results

To understand the relationship between clinical symptoms and IC test kit results, we used the combined results from the two IC test kits. Based on the results of POCT, the samples obtained from the patients were classified into five groups: 1) “A+B−” by SEA-IC and “pdm−” by PDM-IC for seasonal influenza A virus infection; 2) “A−B+” by SEA-IC and “pdm−” by PDM-IC for seasonal influenza B virus infection; 3) “A+B−” by SEA-IC and “pdm+” by PDM-IC for H1N1pdm virus infection; 4) “A−B−” by SEA-IC and “pdm+” by PDM-IC for possible H1N1pdm virus infection; and 5) “A−B−” by SEA-IC and “pdm−” by PDM-IC for the absence of influenza virus infection. Analysis of the relationship between clinical symptoms and POCT results revealed that there was a significant difference between the symptoms in influenza-positive and influenza-negative patients (Table 3). For example, 53.1% of influenza-positive patients versus 44.2% of influenza-negative patients (P = 0.039) presented with an upper respiratory tract inflammation; 27.1% of influenza-positive versus 19.0% of influenza-negative patients (P = 0.024) presented with muscular pain; 3.7% of influenza-positive versus 23.0% of influenza-negative patients presented with pharynx pain (P = 0.000); 29.7% of influenza-positive versus 15.6% of influenza-negative patients presented with a wet cough (P = 0.000); and 35.2% of influenza-positive versus 13.0% of influenza-negative patients presented with a dry cough (P = 0.000).

**Table 3:** Clinical symptoms and pre-existing diseases in study participants.

Symptom/pre-existing disease		Seasonal influenza A virusA+B−, pdm−(n = 46)	Seasonal influenza B virusA−B+, pdm−(n = 19)	H1N1pdmA+B−, pdm+(n = 196)	Possible H1N1pdmA−B−, pdm+(n = 12)	Positive seasonalA/B and H1N1pdm(n = 273)	NegativeA−B−, pdm−(n = 269)
Fever		44 (95.7%)	18 (94.7%)	196 (100%)	12 (100%)	270 (98.9%)	264 (98.1%)
Upper respiratoryinflammation		33 (71.7%)	8 (42.1%)	99 (50.5%)	5 (41.7%)	145 (53.1%)	119 (44.2%)
Lower respiratory	Pneumonia	2 (4.3%)	1 (5.3%)	20 (10.2%)	1 (8.3%)	24 (8.8%)	17 (6.3%)
inflammation	Asthma	0 (0%)	0 (0%)	1 (0.5%)	0 (0%)	1 (0.4%)	0 (0%)
Cough	Wet	15 (32.6%)	6 (31.6%)	57 (29.1%)	3 (25.0%)	81 (29.7%)	42 (15.6%)
	Dry	13 (28.3%)	4 (21.1%)	75 (38.3%)	4 (33.3%)	96 (35.2%)	35 (13.0%)
Arthritis		22 (47.8%)	5 (26.3%)	100 (51.0%)	5 (41.7%)	132 (48.4%)	117 (43.5%)
Muscular pain		11 (23.9%)	3 (15.8%)	58 (29.6%)	2 (16.7%)	74 (27.1%)	51 (19.0%)
Diarrhea		4 (8.7%)	1 (5.3%)	2 (1.0%)	0 (0%)	7 (2.6%)	13 (4.8%)
Vomiting or nausea		2 (4.3%)	1 (5.3%)	9 (4.6%)	1 (8.3%)	13 (4.8%)	16 (5.9%)
Malaise		6 (13.0%)	2 (10.5%)	17 (8.7%)	5 (41.7%)	30 (11.0%)	44 (16.4%)
Pharynx pain		1 (2.2%)	0 (0%)	9 (4.6%)	0 (0%)	10 (3.7%)	62 (23.0%)
Abdominal pain		1 (2.2%)	0 (0%)	2 (1.0%)	0 (0%)	3 (1.1%)	5 (1.9%)
Headache		3 (6.5%)	1 (5.3%)	24 (12.2%)	0 (0%)	28 (10.3%)	21 (7.8%)
Nasal discharge		1 (2.2%)	0 (0%)	11 (5.6%)	0 (0%)	12 (4.4%)	12 (4.5%)
Hypertension		0 (0%)	0 (0%)	2 (1.0%)	1 (8.3%)	3 (1.1%)	7 (2.6%)
Diabetes		0 (0%)	0 (0%)	1 (0.5%)	0 (0%)	1 (0.4%)	1 (0.4%)
Asthma		2 (4.3%)	0 (0%)	5 (2.6%)	0 (0%)	7 (2.6%)	2 (0.7%)
Renal disease		0 (0%)	0 (0%)	0 (0%)	0 (0%)	0 (0%)	1 (0.4%)
Dialysis		0 (0%)	0 (0%)	0 (0%)	0 (0%)	0 (0%)	0 (0%)
Allergy		0 (0%)	1 (5.3%)	3 (1.5%)	0 (0%)	4 (1.5%)	2 (0.7%)
Pregnancy		1 (2.2%)	0 (0%)	0 (0%)	0 (0%)	1 (0.4%)	0 (0%)

No significant differences were observed in terms of other symptoms, such as fever, lower respiratory tract inflammation, arthralgia, or diarrhea. In particular, most of the patients with influenza-like syndrome who were enrolled in this study presented with a high fever, and there was no significant difference (P = 0.463) between patients diagnosed as influenza-positive (98.9%) versus influenza-negative (98.1%). Table 3 illustrates that upper respiratory tract inflammation was discerned more frequently in patients infected with seasonal influenza A virus (“A+B−” by SEA-IC and “pdm−” by PDM-IC, 71.7%), compared with other influenza virus-positive groups (“A−B+” by SEA-IC and “pdm−” by PDM-IC, 42.1%; “A+B−” by SEA-IC and “pdm+” by PDM-IC, 50.5%; and “A−B−” by SEA-IC and “pdm+” by PDM-IC, 41.7%) (P = 0.006). Moreover, malaise was documented more frequently in possible H1N1pdm-positive patients (“A−B−” by SEA-IC and “pdm+” by PDM-IC, 41.7%) relative to the other influenza virus-positive groups (8.7–13.0%) or influenza-negative patients (16.4%) (P = 0.034) (Table 3).

### Anti-viral Drug Prescription

**Figure 3:**
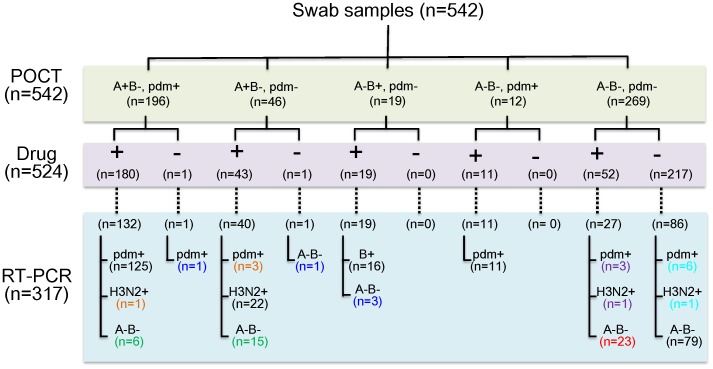
Summarized results for the sensitivity and specificity of the SEA-IC and PDM-IC test kits. First, a total of 542 swab samples were classified into five groups according to the results of POCT. Of these, anti-viral drug prescription information was available for 524 samples. These 524 samples were further classified into two groups: “+” for patients with anti-viral drug prescriptions and “−” for patients without. RT-PCR analysis was performed for 317 of the 524 samples. Numbers in green indicate false-positive IC test kit cases upon RT-PCR analysis, where the clinicians prescribed drugs according to the results of the IC kits. Numbers in blue indicate false-positive IC kit cases, but drugs were not prescribed. Numbers in red indicate negative IC kit cases that were confirmed by RT-PCR, where the clinicians prescribed drugs. Numbers in purple indicate false-negative IC kit cases upon RT-PCR analysis, where the clinicians prescribed drugs. Numbers in light blue indicate false-negative IC kit cases, where drugs were not prescribed although these cases tested positive by RT-PCR. Numbers in black indicate correctly diagnosed cases according to the results of POCT. Numbers in orange indicate that the RT-PCR analysis found detection errors in the IC kit results regarding the detection of H1N1pdm to H3N2 or H3N2 to H1N1pdm.

As shown in Figure 3 and described above, a total of 542 swab samples were subjected to two IC test kits for POCT and stratified into five groups according to the type of virus infection. To understand the relationship between anti-viral drug prescription and the IC test kit results, we used the combined results of the two IC test kits. No information was available for 18 of these samples regarding drug prescription. Therefore, the remaining 524 samples were further grouped according to the presence or absence of drug prescription. Of these, 317 samples were subjected to RT-PCR analysis, 229 collected from individuals who received anti-influenza drug prescriptions (oseltamivir to 154 patients, zanamivir to 63 patients, laninamivir to 8 patients, and peramivir to 4 patients as shown in Table S2) based on POCT results and patient responses to questionnaires, and 88 from individuals who did not (Figure 3).

Of 179 cases confirmed as influenza virus-positive by RT-PCR except 11 cases of “A−B−, pdm+” by the IC test kit results, 95.5% (171/179) were appropriately prescribed anti-influenza drugs based on positive IC test results. However, 8 influenza virus-positive patients out of 179 did not receive prescriptions due to negative IC kit results. An additional 47 cases were prescribed drugs despite influenza virus-negative RT-PCR results due to 18 false-positive SEA-IC kit results, six false-positive results stemming from both kits, and 23 true negative results stemming from both kits. On the other hand, 27 patients at six clinics were prescribed anti-viral drugs even though they tested negative for influenza virus by POCT. Of these 27 patients, three cases of H1N1pdm infection and one case of H3N2 infection were subsequently detected by RT-PCR. Importantly, seven patients who did not receive drug prescriptions because of negative IC results later tested positive for H1N1pdm (six cases) or H3N2 (one case) by RT-PCR.

## Discussion

The present study evaluated a newly-developed PDM-IC kit for its ability to expedite POCT for the detection of H1N1pdm. The study employed 542 duplicate swab samples collected during the 2010/2011 winter influenza season in one ward of Osaka city and two types of IC test kits, the previously-developed SEA-IC kit and the newly-developed PDM-IC kit. Comparison of the data with those obtained by RT-PCR analysis using the same samples revealed that the PDM-IC kit was significantly more sensitive and specific for H1N1pdm than the SEA-IC kit.

The establishment of POCT as a widely accepted method for the diagnosis of influenza depends on physician access to commercially available rapid diagnostic test kits. POCT can easily be performed with such kits within minutes for the detection of seasonal influenza A and B viruses, and the obtained results generally show quite high sensitivity and specificity for these viruses. However, detection of H1N1pdm by test kits that have been developed for seasonal influenza A and B viruses is unreliable, with comparatively low sensitivity and specificity [18], [19], [20], [21], [22], [23], [24], [25], [26]. Therefore, several IC test kits have recently been developed for the selective diagnosis of H1N1pdm [28], [28], [30,31].

In this regard, Choi et al. [30] reported 44.0% sensitivity and 99.9% specificity for the SD Bioline Influenza Antigen Test® (The Clinical Usefulness of the SD Bioline Influenza Antigen Test® for Detecting the 2009 Influenza A (H1N1) Virus). Kawachi et al. [29] similarly reported 73.0% sensitivity and 97.9% specificity for their influenza A H1N1 2009 virus test kit. Our previous study revealed 85.5% sensitivity and 100% specificity for a PDM-IC test kit employed using a total of 42 and 126 swab samples collected at one clinic in Osaka before and after the appearance of H1N1pdm, respectively [27]. The current study expanded on our previous results by comparing the efficacy of the PDM-IC and SEA-IC test kits for the diagnosis of H1N1pdm, and also by using a much larger number of swab samples collected at 13 clinics after the appearance of H1N1pdm. The 542 samples contained both influenza Aand B viruses collected after the appearance of H1N1pdm, whereas the previous study employed only H1N1pdm-confirmed samples collected after the appearance of H1N1pdm.

The results presented herein, using 332 swab samples further analyzed by RT-PCR, showed 92.5% sensitivity and 95.3% specificity for the PDM-IC kit, relative to 89.2% sensitivity and 80.5% specificity for the SEA-IC kit. The high sensitivity and specificity of this and other newly-developed IC test kits for the rapid diagnosis of H1N1pdm can potentially be attributed to the characteristics of the monoclonal antibodies and the cut-off points chosen by the kit development company/personnel. In other words, the company that develops each kit can adjust the sensitivity and specificity of the kit according to its intended use. Users of these test kits should therefore be educated to select the type of kit most appropriate for their needs.

An advantage of the PDM-IC test kit described in this study is that it could be employed with the same swab samples as the SEA-IC kit. Therefore, we were able to obtain results from both test kits using the same samples at the same time. Thus, the PDM-IC kit can be used as a POCT tool to supplement SEA-IC kit data in clinics. Comparison of the data obtained at 13 individual clinics showed significant correlations regarding sensitivity, specificity, and NPV between the SEA-IC kit and the PDM-IC kit. This indicated that the corresponding scores of the IC kits were most likely affected by the skills of the medical and co-medical staff at each clinic. Therefore, increased application of rapid diagnosis test kits for infectious diseases necessitates training in their proper use, and not just selection of the appropriate test kit.

No apparent differences were observed between different age groups in terms of the results yielded by the PDM-IC kit, while some inter-age differences were detected with the SEA-IC kit. Of note, the accuracy of the results of the SEA-IC kit apparently deteriorated at ∼3 days after the onset of clinical symptoms. Similarly, a previous report showed that the sensitivity of another IC test kit for H1N1pdm abruptly declined when using the swab samples collected from patients on the third day after the onset of fever [27]. These results are reasonable given that the number of virus particles excreted by a patient generally decreases at 3–4 days after the onset of symptoms [32]. Nevertheless, the current PDM-IC kit demonstrated high sensitivity when used with samples collected on day third or more days versus day 1 and day 2, further demonstrating the reliability of the kit.

Several cases (12/542 = 2.2%) in this study gave inconsistent results when analyzed with the two different IC test kits. In addition, the IC kit results and RT-PCR results were inconsistent for 14.2% (47/332) of the cases. Although both kits yielded several false-negatives and false-positives, overall, the PDM-IC kit demonstrated better specificity for H1N1pdm than the SEA-IC kit. IC kit-derived false-negatives (IC kit-negative, but RT-PCR-positive) might be explained by the lower sensitivity of the IC kit compared with RT-PCR. On the other hand, there were several IC kit-derived false-positives (IC kit-positive, but RT-PCR-negative). One possible explanation for their occurrence might be the fact that viral antigens might persist even after virus particles had been removed from body. Alternatively, the monoclonal antibody employed by the kit might react with non-specific antigens derived from the host or from other pathogens. When the samples that gave inconsistent IC kit versus RT-PCR were examined by VI, several cases were observed that tested positive by VI, but negative by RT-PCR, especially for H1N1pdm. Furthermore, several additional cases tested negative by VI, but positive by RT-PCR, especially in H3N2. This indicates the possible difficulty of PCR amplification of target H1N1pdm DNA in the former case and of VI of H3N2 in the latter case. Regardless of the cause, further improvements in IC kits are required because false-negative and false-positive results lead to misdiagnosis upon POCT.

A total of 305 of 542 patients were treated with anti-viral drugs at 13 clinics in the present study. When the RT-PCR results were examined for 229 of these 305 drug-subscribed patients, we found that 23 individuals were given drugs even though they tested negative for the influenza virus by both IC test kits and RT-PCR. The drugs were dispensed because of patient responses to questionnaires regarding typical clinical symptoms of influenza, influenza infection in close relationships, friends, and/or colleagues, or an affected individual’s desire to receive anti-viral drugs within the first day of fever occurrence, typically at a clinic that is open over the weekend. The latter case is thought by the conditions for influenza virus detection in such febrile patients might simply be too early, leading to false-negative results upon POCT.

Soon after the emergence of H1N1pdm, the virus was thought to be sensitive to oseltamivir (Tamiflu); however, several recent papers report an increasing incidence of drug-resistant H1N1pdm strains [5]–[8]. In 2004, an oseltamivir-resistant H3N2 strain was frequently detected in infants who were treated with the drug in Japan. This indicates a correlation between drug dosage and the manifestation of resistant strains [33]. Therefore, the use of an IC test kit with high sensitivity and specificity for H1N1pdm, together with a physician preference for IC test kit data instead of clinical influenza-like symptoms and patient responses to questionnaires, are strongly recommended prior to the prescription of anti-influenza drugs. Moreover, special attention must be paied to the fact that anti-viral drugs (e.g., oseltamivir) are used more heavily in Japan than in any other country in the world for the treatment and even prophylaxis of influenza. Although such drug use appears to have significantly reduced lethality due to H1N1pdm infections in Japan compared with other countries [16], [17], the extensive use of anti-viral medication increases the risk of resultant drug-resistant influenza virus variants. The current study revealed that, since the appearance of H1N1pdm in 2009, anti-viral prescription practices of clinics in Japan are not always in accordance with the data afforded by IC kits. Therefore, the information provided by this study regarding the high reliability of concomitant use of SEA-IC and PDM-IC kits for the rapid diagnosis of influenza may lead to reduced prescription of anti-viral drugs, as well as diminished emergence of drug-resistant virus variants.

The future global status of influenza viruses, including the evolution of H1N1pdm and/or the appearance of new pandemic strains, is largely unknown. Zhu et al. reported a new reassortant virus in swine in 2010 [34], and the Centers for Disease Control and Prevention reported a new case of swine-origin influenza A virus H3N2 in 2011 [35]. Thus, future surveillance of influenza viruses, followed by the rapid development of new IC kits for the diagnosis of such newly emerged viruses, is extremely important to human welfare. Because the PDM-IC test kit introduced in this study does not require any specialized equipment, it can be use both at the bedside and for field surveillance. Hence the PDM-IC kit and related kits are anticipated to be important tools for the identification and control of influenza viruses.

## Supporting Information

Table S1 Duration of days after disease onset categorized by each age group.
(DOCX)

Table S2 Antiviral drug prescription information from 13 clinics for influenza virus-positive cases confirmed by RT-PCR.
(DOCX)
