# Supplementary material for: Reliability of a Newly-Developed Immunochromatography Diagnostic Kit for Pandemic Influenza A/H1N1pdm Virus: Implications for Drug Administration
Source: PLoS One. 2012 Nov 30;7(11):e50670. doi: 10.1371/journal.pone.0050670 (PMC3511324; doi:10.1371/journal.pone.0050670)
Supplement: Table S1 — Duration of days after disease onset categorized by each age group. (DOCX) [file pone.0050670.s001.docx]

| Age (years) | | <10 | 10–19 | 20–29 | 30–39 | 40–49 | 50–59 | ≥60 |
| --- | --- | --- | --- | --- | --- | --- | --- | --- |
| Days after disease onset  (days) | Total  (Cases) | 0.65±0.69  (34) | 1.05±1.17  (60) | 1.74±1.63  (73) | 1.19±0.77  (64) | 1.46±1.72  (41) | 1.40±1.32  (25) | 1.53±0.83  (15) |
|  | False-positive  (Cases; %^1)^) | 0.75±0.96  (4; 11.7%) | 0.67±0.58  (3; 5.0%) | 2.00±1.65  (12; 16.4%) | 1^2)^  (1; 1.6%) | -  (0; 0.0%) | 0^2)^  (1; 4.0%) | 2.50±0.71  (2; 13.3%) |
|  | False-negative  (Cases; %^1)^) | -  (0; 0.0%) | 1.00±1.41  (2; 3.3%) | 1.71±0.76  (7; 9.6%) | 1.00+0.00  (3; 4.7%) | 1.67±1.15  (4; 9.8%) | 1.00±0.00  (4; 16.0%) | -  (0; 0.0%) |

1) Percentage of false-positive/negative cases.

2) The standard deviation was not calculated due to a sample size of one.
