# Supplementary material for: Reliability of a Newly-Developed Immunochromatography Diagnostic Kit for Pandemic Influenza A/H1N1pdm Virus: Implications for Drug Administration
Source: PLoS One. 2012 Nov 30;7(11):e50670. doi: 10.1371/journal.pone.0050670 (PMC3511324; doi:10.1371/journal.pone.0050670)
Supplement: Table S2 — Antiviral drug prescription information from 13 clinics for influenza virus-positive cases confirmed by RT-PCR. (DOCX) [file pone.0050670.s002.docx]

|  |  |  |  | Clinic | | | | | | | | | | | | |
| --- | --- | --- | --- | --- | --- | --- | --- | --- | --- | --- | --- | --- | --- | --- | --- | --- |
| Drug | SEA-IC | PDM-IC | RT-PCR | A | B | C | D | E | F | G | H | I | J | K | L | M |
| None | A-B- | pdm- | - | 2 | 0 | 22 | 7 | 4 | 4 | 1 | 12 | 0 | 6 | 2 | 6 | 13 |
|  | A-B- | pdm - | H1N1pdm | 0 | 1 | 0 | 3 | 0 | 0 | 1 | 0 | 0 | 1 | 0 | 0 | 0 |
|  | A-B- | pdm - | H3N2 | 0 | 0 | 0 | 0 | 0 | 0 | 1 | 0 | 0 | 0 | 0 | 0 | 0 |
|  | A+B- | pdm - | - | 0 | 0 | 0 | 0 | 0 | 0 | 0 | 0 | 0 | 0 | 0 | 1 | 0 |
|  | A+B- | pdm + | H1N1pdm | 0 | 0 | 0 | 0 | 1 | 0 | 0 | 0 | 0 | 0 | 0 | 0 | 0 |
| Oseltamivir | A-B- | pdm - | - | 2 | 3 | 2 | 5 | 0 | 0 | 0 | 1 | 0 | 0 | 0 | 0 | 1 |
|  | A-B- | pdm - | H1N1pdm | 0 | 1 | 0 | 1 | 0 | 0 | 0 | 0 | 0 | 0 | 0 | 0 | 0 |
|  | A-B- | pdm - | H3N2 | 0 | 1 | 0 | 0 | 0 | 0 | 0 | 0 | 0 | 0 | 0 | 0 | 0 |
|  | A-B- | pdm + | H1N1pdm | 0 | 0 | 0 | 2 | 0 | 0 | 0 | 1 | 0 | 1 | 0 | 1 | 2 |
|  | A+B- | pdm - | - | 0 | 0 | 0 | 0 | 0 | 0 | 1 | 2 | 3 | 0 | 1 | 2 | 1 |
|  | A+B- | pdm - | H1N1pdm | 0 | 0 | 0 | 0 | 0 | 0 | 0 | 1 | 0 | 1 | 0 | 0 | 1 |
|  | A+B- | pdm - | H3N2 | 0 | 0 | 2 | 0 | 3 | 0 | 0 | 0 | 2 | 2 | 1 | 3 | 2 |
|  | A+B- | pdm + | - | 0 | 0 | 1 | 0 | 1 | 0 | 2 | 0 | 0 | 0 | 0 | 1 | 0 |
|  | A+B- | pdm + | H1N1pdm | 4 | 0 | 15 | 8 | 2 | 0 | 14 | 3 | 5 | 2 | 11 | 17 | 4 |
|  | A-B+ | pdm - | - | 0 | 0 | 0 | 0 | 0 | 0 | 0 | 1 | 0 | 1 | 0 | 0 | 0 |
|  | A-B+ | pdm - | B | 2 | 0 | 4 | 0 | 0 | 0 | 1 | 2 | 0 | 0 | 1 | 0 | 0 |
| Zanamivir | A-B- | pdm - | - | 0 | 0 | 1 | 3 | 0 | 0 | 0 | 0 | 0 | 0 | 0 | 0 | 1 |
|  | A-B- | pdm - | H1N1pdm | 1 | 0 | 0 | 0 | 0 | 0 | 0 | 0 | 0 | 0 | 0 | 0 | 0 |
|  | A-B- | pdm + | H1N1pdm | 0 | 0 | 0 | 2 | 0 | 0 | 0 | 0 | 0 | 0 | 0 | 0 | 2 |
|  | A+B- | pdm - | - | 0 | 0 | 0 | 0 | 0 | 0 | 0 | 1 | 1 | 0 | 0 | 0 | 2 |
|  | A+B- | pdm - | H3N2 | 0 | 0 | 4 | 1 | 0 | 0 | 0 | 0 | 2 | 0 | 0 | 0 | 0 |
|  | A+B- | pdm + | - | 0 | 0 | 1 | 0 | 0 | 0 | 0 | 0 | 0 | 0 | 0 | 0 | 0 |
|  | A+B- | pdm + | H1N1pdm | 1 | 0 | 2 | 25 | 0 | 1 | 0 | 3 | 2 | 0 | 0 | 0 | 1 |
|  | A+B- | pdm + | H3N2 | 0 | 0 | 0 | 1 | 0 | 0 | 0 | 0 | 0 | 0 | 0 | 0 | 0 |
|  | A-B+ | pdm - | - | 0 | 0 | 1 | 0 | 0 | 0 | 0 | 0 | 0 | 0 | 0 | 0 | 0 |
|  | A-B+ | pdm - | B | 1 | 0 | 0 | 1 | 0 | 0 | 0 | 1 | 0 | 1 | 0 | 0 | 0 |
| Peramivir | A+B- | pdm + | H1N1pdm | 0 | 3 | 0 | 0 | 0 | 0 | 0 | 0 | 0 | 0 | 0 | 0 | 1 |
| Laninamivir | A-B- | pdm - | - | 0 | 1 | 0 | 0 | 0 | 0 | 0 | 0 | 0 | 0 | 0 | 0 | 3 |
|  | A+B- | pdm - | - | 0 | 0 | 0 | 0 | 0 | 0 | 0 | 0 | 0 | 0 | 0 | 0 | 1 |
|  | A+B- | pdm + | H1N1pdm | 0 | 0 | 0 | 0 | 0 | 0 | 0 | 0 | 0 | 0 | 0 | 0 | 1 |
|  | A-B+ | pdm - | B | 2 | 0 | 0 | 0 | 0 | 0 | 0 | 0 | 0 | 0 | 0 | 0 | 0 |
